# Remission of Cognitive Deficits in Parkinson's Disease: Recovery from a Nonamnestic Mild Cognitive Impairment or Psychiatric Symptoms Remission?

**DOI:** 10.1155/2012/427698

**Published:** 2012-11-05

**Authors:** Jonas Jardim de Paula, Marco Túlio Gualberto Cintra, Débora Marques Miranda, Maria Aparecida Camargos Bicalho, Edgar Nunes Moares, Leandro Fernandes Malloy-Diniz

**Affiliations:** ^1^Laboratório de Investigações Neuropsicológicas (LIN), Universidade Federal de Minas Gerais, 31270-901 Belo Horizonte, MG, Brazil; ^2^INCT de Medicina Molecular, Faculdade de Medicina, Universidade Federal de Minas Gerais, Avenida Alfredo Balena 190, 30130-100 Belo Horizonte, MG, Brazil; ^3^Instituto Jenny de Andrade Faria de Atenção à Saúde do Idoso, Hospital das Clínicas, Universidade Federal de Minas Gerais, 31270-901 Belo Horizonte, MG, Brazil; ^4^Departamento de Clínica Médica, Faculdade de Medicina, Universidade Federal de Minas Gerais, 31270-901 Belo Horizonte, MG, Brazil; ^5^Departamento de Saúde Mental, Faculdade de Medicina, Universidade Federal de Minas Gerais, 31270-901 Belo Horizonte, MG, Brazil

## Abstract

Mild cognitive impairment is a clinical condition more frequent in patients with Parkinson's disease than in general population. The nonamnestic presentations, usually characterized by executive dysfunction, are most prevalent. We present a case report of a Parkinson's disease patient diagnosed with nonamnestic mild cognitive impairment that showed complete remission of cognitive symptoms after one year. We discuss the possible causes for the remission, focusing on the treatment of medical conditions such as a major depressive episode and vitamin B12 deficiency, in addition to the change of pharmacological treatment. In a third assessment, cognitive performance remained normal. The case report highlights the importance of controlling clinical comorbidities on the assessment and followup of mild cognitive impairment, especially on Parkinson's disease.

## 1. Introduction

The concept of mild cognitive impairment (MCI) in Parkinson's disease refers to a cognitive disorder where the subject shows an objective impairment in one or more cognitive domains and lack of or only mild functional impairment [1]. Due to the absence of uniformity in diagnostic criteria, there is little agreement in the literature on what subtype of MCI (amnestic × non-amnestic, single domain × multiple domain) is the most frequent in Parkinson's disease.

The often used criteria for the diagnosis of MCI take into consideration the cognitive performance of the subject and establish that the patient should have a performance in terms of 1.5 standard deviations below the population mean of those subjects with the same age and years of formal education [2]. These same criteria are recommended considering MCI diagnosis in Parkinson's disease [1]. Nonetheless, there is evidence that MCI is undiagnosed in Parkinson's disease, and this fact can be related to the excessive use of screening tools, which are less sensitive to the cognitive decline observed in this population [3].

In Parkinson's disease patients, MCI is more prevalent than in general population, affecting about 30% of the individuals, and the nonamnestic executive is the most commonly diagnosed form [4, 5]. The conversion rate to dementia is about 70% in four years [6]. There is little evidence that MCI is reversible considering data from population and longitudinal studies [7]. Nonetheless, these data are from studies in general population and in those subjects with conversion to dementia syndromes. Mamikonyan and colleagues [3] suggest that in Parkinson's disease some causes of cognitive impairment are reversible, such as daytime sleepiness and psychiatric symptoms. In this report, we present a case of nonamnestic MCI in Parkinson's disease and its remission of cognitive impairments after one year. We thereafter discuss the possible mechanisms underlying this remission.

## 2. Case Description and Clinical Exam

BS is a 53-year-old male, 8 years of formal education, married and retired from his former occupational activities, and Parkinson's disease diagnosed in 1999. In 2010, the patient was referred by his neurologist to the *Instituto Jenny de Andrade Faria de Atenção à Saúde do Idoso*, a secondary public health unit of Belo Horizonte city, for further evaluation. The initial hypothesis questioned by the neurologist was dementia related to Parkinson's disease. 

According to the patient's wife, since 2009 BS had been showing repetitive behavior, psychomotor slowness and inattention. No memory complaints, temporal or topographic disorientation, and language or reasoning impairments were related. The primary complaints initiated after the patient's retirement and beginning of sexual dysfunction. The physical exam showed dyskinesia (of chorea type) on superior limbs and chin. No impairment in basic or instrumental daily life activities was related.

Neuropsychiatric symptoms on the first exam were related. According to the neuropsychiatric inventory [8], signs of delusional thoughts of infidelity (4), depression (1), anxiety (8), euphoria (2), disinhibition (2), and irritability/lability (2) were identified. The most dysfunctional symptom was the delusional thoughts, which were affecting the patient's relationship with his wife. The patient was also diagnosed with a major depressive episode considering the DSM-IV criteria. His serum levels of B12 vitamin were low (114 pg/mL). The magnetic resonance imaging showed signs of diffuse cortical atrophy. On the first assessment, the patient was being treated with Amantadine (300 mg/day), Levodopa/Benserazide (250 mg/day), and B12 vitamin (5000 units/2 times per month).

The patient was submitted to a cognitive screening conducted by the gerontologist. The screening was composed by the Brazilian versions of the Minimental State Exam [9], the Pfeffer functionality index [10], the verbal learning of the CERAD battery [11], the Category Fluency Test [11], and the Clock Drawing Test [12].  Table 1 shows these preliminary results.

The clinical impression was of a nonamnestic MCI secondary to Parkinson's disease, a major depressive episode, B12 vitamin deficiency and erectile dysfunction. Because of the clinical findings, the anticholinergic drugs (Amantadine and Levodopa/Benserazide) were gradually discontinued, and the Pramipexole was raised to 3 mg/day. A 25 mg/day dose of Nortriptyline was initiated, gradually increased to 100 mg/day. The B12 vitamin reposition was maintained. After the clinical exam, the patient was referred to neuropsychological assessment for a better clarification of the cognitive symptoms.

## 3. Neuropsychological Assessment

The patient was examined by a clinical neuropsychologist using a comprehensive protocol several cognitive functions but focusing on executive functioning. The rationale of the neuropsychological assessment used was based on a hierarchical cognitive architecture model [13], aiming at the assessment of global cognitive functioning, memory, language, processing speed, visuospatial abilities, and executive functions. The following tests were used on the assessment. Although the diagnosis was performed before the most recent consensus [1], our guidelines for the MCI diagnosis were very similar to the levels I and II assessment of MCI: a more general impairment on a validated cognitive scale for Parkinson's disease dementia, impairment (at least 1.5 standard deviation below the age/education population reference value) in at least two validated neuropsychological tests for the specific cognitive domain and lack of functional impairment in basic and instrumental daily life activities. 

### 3.1. Cognitive Status and Global Measures

The Dementia Rating Scale (DRS) [14] total score was chosen for global cognitive screening, since this test was well suited for the diagnosis of cognitive impairment in Parkinson's disease [15].

### 3.2. Episodic Memory

The Brazilian version of the Rey Auditory Verbal Learning Test (RAVLT) [16], a complex figure recall [17] and the DRS Memory subscale [14] were used as episodic memory measures. The components A6 (immediate recall), A7 (delayed recall), Total (learning) and recognition (recognition memory) from RAVLT, as well the immediate and delayed recall of the complex figure, and the total score of DRS Memory Subscale were selected as episodic memory measures.

### 3.3. Language

The TN-LIN is a naming test adapted for Brazilian elders [18] that uses stimuli more common to our patients than the other more traditional naming tests do, and it was used for the assessment of language. The Brazilian version of the Token Test [19], a measure of verbal comprehension, was also selected for this assessment.

### 3.4. Processing Speed

We used a “Miniverbal” test for the assessment of this cognitive domain. The 5 Digits Test [20] is a stroop effect test designed to be culture free. It uses a numeric/counting stroop paradigm and assesses automatic attentional process (reading numbers and counting small figures), a simple measure of processing speed [21].

### 3.5. Visuospatial Abilities

The Clock Drawing Test [12] is commonly used for the assessment of constructional abilities, and was selected for the exam. The “Construction” subscale of the dementia rating scale [14], an adapted complex figure copy [17], and the Stick Design Test [22] were also selected as measures of this function.

### 3.6. Executive Functions

The assessment of this multifaceted cognitive domain was carried out focusing on its specific components. The “Initiative/Perseveration” subscale of the DRS was used as a more general measure of executive functioning. The *Planning Skills* were assessed by a modified version of the Tower of London Test which is more sensitive for mild cognitive impairment [23]. *Fluency* was measured by a verbal test, the Category Fluency of “Fruits” [24] and a drawing test, the Modified Five-Point Test [25]. On this last measure, the perseverations and strategy indexes were also evaluated. The 5 Digits Test [21] components of inhibition (time, errors, and executive score) were used as a measure of *Inhibitory Control*. For *Categorization* the subscale “Conceptualization” of the dementia rating scale was selected [14]. Finally, the perseverations of the Modified Five-Points Test and the components of shifting (time, errors, and executive score) of the 5 Digits Test [21] were used as a measure of* Cognitive Shifting*. These conjunct measures provide a comprehensive method for the assessment of executive functions.

**Figure 1:**
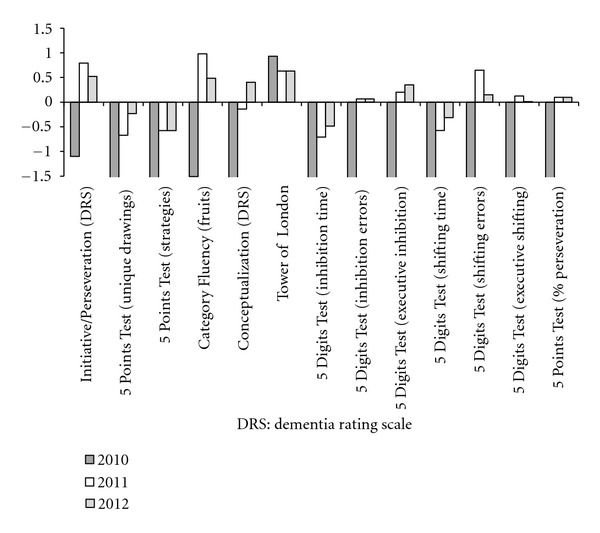
Executive functions profile.

Table 2 shows the assessment results. For better interpretation, all patient raw scores were transformed in *Z* scores based on the Brazilian normative values and corrected by age, formal education, and gender when necessary. Time, errors and perseveration scores were multiplied by −1 for easier analysis. Considering the threshold of 1.5 standard deviations below the normative reference values, the patient shows impaired performance on several executive functions measures, especially those related to fluency, inhibitory control, and cognitive shifting (Figure 1). The impairments in processing speed (about 1 SD below the reference value) could mediate the deficits in inhibitory control, cognitive shifting and fluency tasks; however, the impairment was present even in the nontimed scores, suggesting a loss of speed and efficiency. The data was then discussed by the multidisciplinary team, considering cognitive symptoms presented, its onset and progression, the neuropsychological profile presented, and the biases due to other clinical conditions besides PD which could underlie the cognitive impairment (depression, reduced vitamin B12, and patients pharmacological treatment). The diagnosis of MCI was proposed by the multidisciplinary team, and the patient went for followup.

The patient was reevaluated one year later. There were remission of depressive and psychotic symptoms, normalization of B12 vitamin levels, and good acceptance for the new pharmacological treatment. Cognitive complaints were milder. On this assessment, parallel forms of the neuropsychological tests, when available, were used. The results show a marked cognitive improvement in executive functions and remission of neuropsychiatric symptoms. Based on this data, we considered the remission of the nonamnestic MCI diagnosis. One year later, a new assessment confirmed the stability of cognitive performance. An FDG PET-CT exam performed one week after the last neuropsychological assessment reveals no significant change signs on cerebral cortex, cerebellum, basal ganglia, and thalamus glucose metabolism.

## 4. Discussion

Our findings indicated that after controlling for clinical aspects such as depression, B12 deficiency, and inadequate pharmacological treatment, the cognitive deficit which led to the diagnosis of nonamnestic MCI associated with Parkinson's disease could be remitted. Those three clinical conditions are associated with cognitive impairment and might have contributed for the patient's performance on cognitive tests.

Patients with Parkinson's disease and without dementia usually present cognitive impairment [1, 26], even when more general cognitive measures such as the Minimental State Exam suggests average results [3]. Since the cognitive performance of Parkinson's disease patients follows a nonnormal distribution [27], the use of strictly objective criteria (such as the 1.5 standard deviation in only one test) could lead to misguided results, demanding a more comprehensive diagnostic approach. Usually, the MCI diagnosis is performed after subjective complaint of cognitive changes. For our patient, only 10 years after the diagnosis of Parkinson's disease, the subjective cognitive complaints were related, leading to the cognitive assessment. On the first assessment, the diagnosis was performed after the convergent finding of executive impairment on different neuropsychological tests, even with the normal Minimental State result, lack of impairment on basic and instrumental daily life activities, no signs of dementia according to the standard diagnosis procedures.

Anticholinergic drugs as the tricyclic antidepressants, antipsychotics, antihistamines, and antiparkinsonians usually show a range of side effects in late life, such as falls, visual blurring, delirium, and more pronounced neuropsychiatric symptoms [28]. A prospective study [29] indicated that anticholinergic drugs may impair attentional processes but are not associated with progressive cognitive decline. In a transversal study, other authors [30] related a selective impairment on verbal episodic memory. Impaired verbal episodic memory is the most common cognitive impairment due to use of anticholinergic drugs, although there is no consensus about this pattern. The impairment is not sufficient to cause dementia, but it may be related to mild and reversible cognitive deficits. In our patient, the change from Amantadine and Levodopa/Benserazide to Nortriptyline and Pramipexole was associated with better cognitive performance on executive functions tests, even when we consider the anticholinergic effect of these new drugs [31]. 

The association between B12 vitamin and cognition still remains unclear. Although some studies associated this nutrient with cognition [32], others prove to be inconclusive [33]. A recent study [34] suggests that a specific marker of B12 deficiency (Methylmalonate) might affect cognitive performance, having brain volume atrophy, changes in white matter integrity, and cerebral infarcts as mediators. Blasko and colleagues [35] showed that B12 serum levels are not related to cognitive performance, but the supplementation of B12 associated with folic acid might reduce the conversion rate from MCI to dementia, probably by the reduction of homocysteines. These results, however, are controversial, since other studies [36] did not show convergent findings. It is not clear how B12 vitamin deficit in our patient might be associated with the observed cognitive impairment, but it is a hypothesis for the MCI remission.

It is important to note that the cognitive improvement presented by the patient could be associated with the remission of depressive symptoms due to pharmacological therapy. The role of depressive symptoms as moderators of cognitive performance is well established in neuropsychology [37, 38]. Although the underlying mechanism of how depression associates with cognitive impairment is not completely clear, the changes on mesial temporal structures such as the hippocampus and amygdala associated with the disruption of frontostriatal connections may underlie the frequently related executive functions and episodic memory impairments in depression [38]. Recently, Sexton and colleagues [39] suggested that the executive dysfunction and the slowness of processing speed, two core features of cognitive impairment in depression, may mediate the deficits in other cognitive domains. In this regard, depression treatment may have promoted a better cognitive performance in our patient. A recent review on MCI in Parkinson's disease [40] described the multiple pathogenic mechanisms for cognitive impairment, considering a degenerative, a vascular and a psychiatric component. Even in the presence of Parkinson's disease, our patient also had a major depressive episode diagnosis, which, at least partially, may be related to cognitive impairment.

## 5. Conclusion

Our case report is an example of remission of a cognitive deficit in a nonamnestic MCI associated with Parkinson's disease. Considering the multifactorial etiology of MCI, our results show the relevance of a comprehensive assessment and followup in these cases. Furthermore, our results highlight areas for future studies such as the followup of patients with MCI related to Parkinson's disease after pharmacological interventions and the role of psychiatric disorders as moderators in cognitive deficits in this disorder. 

## Figures and Tables

**Table 1:** Patient's cognitive screening (January 2010).

Measure	Patient's score	Cut-off
Minimental state exam	26	22
Clock drawing test	4	3
Category fluency (animals)	15	11
Verbal learning (total words)	20	13
Verbal learning (recall)	6	4
Verbal learning (recognition)	10	7
Pfeffer index	0	5

**Table 2:** Neuropsychological assessment.

	First assessment (Jan/2010)		Second assessment (Jan/2011)		Third assessment (Jan/2012)		Reference value	
	Raw score	*Z* score	Raw score	*Z* score	Raw score	*Z* score	Mean	SD
Screening tests								
MMSE	26	−1,1	29	0,4	29	0,4	28,3	2,0
DRS	121	−1,9*	137	0,6	137	0,6	133,2	6,3

Memory								
Memory (DRS)	25	1,3	25	1,3	25	1,3	22,4	2,0
RAVLT (immediate recall)	11	0,2	14	1,3	14	1,3	10,4	2,7
RAVLT (delayed recall)	9	−0,3	13	1,3	13	1,3	9,8	2,4
RAVLT (recognition)	13	0,4	14	0,9	14	0,9	12,1	2,1
Complex figure (immediate recall)	48	−0,8	59	−0,1	65	0,3	60,0	16,0
Complex figure (delayed recall)	47	−0,6	75	1,4	70	1,1	55,0	14,0

Language								
Token test	30	0,0	35	1,3	33	0,8	30,0	4,0
TN-LIN (naming)	64	0,7	64	0,7	62	0,3	60,2	5,7

Processing speed								
5 Digits test (reading)	30	−1,2	27	−0,6	23	0,3	24,3	4,9
5 Digits test (counting)	33	−1,4	28	−0,2	26	0,3	27,3	4,2

Visuospatial								
Constructional praxis (DRS)	6	0,3	6	0,3	6	0,3	5,8	0,8
Stick design test	12	0,3	12	0,3	12	0,3	11,9	0,5
Clock drawing	4	−0,1	4	−0,1	4	−0,1	4,1	1,3
Complex figure copy	63	−1,8*	83	−0,5	85	−0,3	90,0	15,0

Executive functions								
Global measure								
Initiative/perseveration (DRS)	30	−1,1	37	0,8	36	0,5	34,1	3,7

Fluency								
5 Points test (unique drawings)	19	−1,6*	23	−0,7	25	−0,2	26,1	4,5
5 Points test (strategies)	0	−1,6*	4	−0,6	4	−0,6	6,22	3,84
Category fluency (fruits)	12	−1,5*	17	1,0	16	0,5	15,0	2,0

Categorization								
Conceptualization (DRS)	26	−2,6*	35	−0,1	37	0,4	35,5	3,7

Planning								
Tower of London	39	0,9	37	0,6	37	0,6	32,8	6,7

Inhibitory control								
5 Digits test (inhibition time)	59	−2,7*	41	−0,7	39	−0,5	34,6	9,0
5 Digits test (inhibition errors)	7	−3,3*	2	0,1	2	0,1	2,1	1,5
5 Digits test (executive inhibition)	29	−2,0*	14	0,2	13	0,4	15,4	6,8

Cognitive shifting								
5 Digits test (shifting time)	78	−2,8*	52	−0,6	49	−0,3	45,4	11,5
5 Digits test (shifting errors)	11	−3,4*	3	0,7	4	0,2	4,3	2
5 Digits test (executive shifting)	48	−2,5*	25	0,1	26	0,0	26,1	8,7
5 Points test (% perseveration)	19	−2,8*	4	0,1	4	0,1	4,5	5,2

MMSE: minimental state exam, DRS: dementia rating scale, and *equal or below the 1.5 SD threshold.
